# The Effects of Wenxin Keli on P-Wave Dispersion and Maintenance of Sinus Rhythm in Patients with Paroxysmal Atrial Fibrillation: A Meta-Analysis of Randomized Controlled Trials

**DOI:** 10.1155/2013/245958

**Published:** 2013-12-04

**Authors:** Yu Chen, Shaoping Nie, Hai Gao, Tao Sun, Xiaoqiu Liu, Fei Teng, Yanhui Xing, Wen Chen, Zhenpeng Zhang, Yonghong Gao, Jie Wang, Yanwei Xing, Hongcai Shang

**Affiliations:** ^1^Guang'anmen Hospital, Chinese Academy of Chinese Medical Sciences, Beijing 100053, China; ^2^Shenyang Pharmaceutical University, Shenyang, Liaoning 110016, China; ^3^Beijing An Zhen Hospital of the Capital University of Medical Sciences, Beijing 100029, China; ^4^Institute of Information on Traditional Chinese Medicine, Academy of Chinese Medical Sciences, Beijing 100700, China; ^5^The Key Laboratory of Chinese Internal Medicine of the Ministry of Education, Dongzhimen Hospital Affiliated to Beijing University of Chinese Medicine, Beijing 100700, China; ^6^Tianjin University of Traditional Chinese Medicine, Tianjin 300193, China

## Abstract

*Objective*. To evaluate the beneficial and adverse effects of Wenxin Keli (WXKL), alone or combined with Western medicine, on P-wave dispersion (Pd) and maintenance of sinus rhythm for the treatment of paroxysmal atrial fibrillation (PAF). *Methods*. Seven major electronic databases were searched to retrieve randomized controlled trials (RCTs) designed to evaluate the clinical effectiveness of WXKL, alone or combined with Western medicine, for PAF, with Pd or maintenance rate of sinus rhythm as the main outcome measure. The methodological quality of the included studies was assessed using criteria from the Cochrane Handbook for Systematic Review of Interventions, version 5.1.0, and analysed using RevMan 5.1.0 software. *Results*. Fourteen RCTs of WXKL were included. The methodological quality of the trials was generally evaluated as low. The results of meta-analysis showed that WXKL, alone or combined with Western medicine, was more effective in Pd and the maintenance of sinus rhythm, compared with no medicine or Western medicine alone, in patients with PAF or PAF complicated by other diseases. Seven of the trials reported adverse events, indicating that the safety of WXKL is still uncertain. *Conclusions*. WXKL, alone or combined with Western medicine, appears to be more effective in improving Pd as well as maintenance of sinus rhythm in patients with PAF and its complications.

## 1. Introduction

Atrial fibrillation (AF) is the most common arrhythmia contributing to an epidemic of cardiovascular disease that has emerged in the new millennium. It is responsible for considerable morbidity and mortality. The advent of catheter ablation for patients with AF has provided new insights into the relative contribution of AF to left ventricular dysfunction [1]. Paroxysmal atrial fibrillation (PAF) has the tendency to develop into persistent atrial fibrillation and permanent atrial fibrillation, so the longer the duration of AF, the greater the difficulty of the treatment [2]. AF and heart failure (HF) often coexist [3]. AF patients with HF, particularly patients with HF and reduced ejection fraction, experience heavy symptom and hospitalization burdens and have relatively low rates of AF control. So, more studies are needed to identify ways to improve the management and treatment outcomes of this very high-risk patient population.

P-wave dispersion (Pd) and P maximum (Pmax) are new concepts proposed by Dilaveris et al. in 1998 that are simple electrocardiographic markers that can be used for the prediction of idiopathic PAF. The increase of Pd and Pmax is an important indicator of subsequent attacks of PAF and the tendency towards persistent atrial fibrillation [4]. Observational study had investigated the predictive power of P-wave dispersion (PWD) for the incidence of postcardiac surgery AF [5]. It determined that minimum P-wave duration, PWD, and low ejection fraction can be used for patient risk stratification of AF after coronary artery bypass grafting surgery. These years P maximum/P dispersion and high-sensitivity C-reactive protein (hs-C-reactive protein) also have been proposed as useful markers for predicting the history and recurrence of AF [6]. It indicated that subclinical inflammation may be associated with delayed/inhomogeneous atrial activation in hypertensive patients affected by AF.

Despite the fact that the use of radiofrequency and cryoablation has made significant progress, antiarrhythmic drug therapy for AF is still the preferred option for controlling heart rate [7]. The curative effect of pure Western medicine on PAF is still unsatisfactory. During long-term Western medicine treatment, typical side effects accrue [8]. Therefore, it is particularly necessary to explore traditional Chinese medicine for the treatment of PAF and to give full consideration to the role of Chinese medicine in PAF treatment. The clinical achievements of the past 30 years have indicated that integrative medicine, which builds on the combination of both Western medicine and traditional Chinese medicine, has made tremendous contributions for the great rejuvenation of the Chinese nation and human health care [9].

Wenxin Keli (WXKL) is a pure Chinese medicine, developed by Guang'anmen Hospital, Chinese Academy of Chinese Medical Sciences, that has a moderate antiarrhythmic effect. Studies have shown that it can significantly improve patient heart palpitations, chest tightness, shortness of breath, fatigue, insomnia, and other symptoms and that it can have a significant effect on controlling a variety of arrhythmias, right ventricular contractions, atrial premature contractions, AF, and sinus tachycardia. It is safe, reliable, has no side effects, and is appropriate for long-term use [10]. WXKL is reported to be effective in the treatment of atrial and ventricular cardiac arrhythmias. Data provided support for the hypothesis that WXKL, particularly in combination with quinidine, effectively suppresses arrhythmogenesis in an experimental model of Brugada syndrome via inhibition of Ito and indirect adrenergic sympathomimetic effects [11]. The latest study indicated that Wenxin Keli could suppress atrial substrate remodelling after epicardial ganglionic plexi ablation [12].

Currently, WXKL combined with antiarrhythmic drugs, a new integrative medicine therapy, has been widely used as an alternative and effective method for AF in China. A large number of clinical studies reported the clinical effect of WXKL and WXKL combined with antiarrhythmic drugs. And, until now, a large number of randomized controlled trials (RCTs) and case series have been published but have not been evaluated according to the PRISMA systematic review standard. And the predictive value of Pd on PAF in patients treated with WXKL has not been determined. Understanding the effect of WXKL on Pd and maintenance rate of sinus rhythm could be valuable for the management of PAF. Therefore, this study aims to assess the current clinical evidence of WXKL combined with antiarrhythmic drugs for PAF and seeks to identify the relationship between Pd and PAF in patients treated with WXKL and evaluate the efficacy and safety of WXKL for the treatment of PAF.

## 2. Materials and Methods

### 2.1. Database and Search Strategies

The literature search was conducted using the Chinese National Knowledge Infrastructure (CNKI), the Chinese Biomedical Literature Database (CBMdisc), the Chinese Scientific Journal Database (VIP), Wanfang Database, EMbase, PubMed, and the Cochrane Library. The search concluded in May, 2013. Other relative research papers were searched by hand. The following search terms were used individually or in combination: “Wenxin Keli,” “Wenxinkeli,” “Wenxin Granules,” “Wenxin Granule,” “atrial fibrillation,” “auricular fibrillation,” and “randomized controlled trial.” The bibliographies of the included studies were searched for additional references.

### 2.2. Inclusion and Exclusion Criteria

All RCTs of patients with PAF that studied prescriptions based on WXKL, alone or combined with Western medicine, compared with no medicine or Western medicine alone were included. There were no restrictions on language, population characteristics, and publication type. The primary outcome measure was Pd or maintenance of sinus rhythm, and the secondary outcome measure was adverse drug reaction (ADR). Duplicated publications reporting the same groups of participants were excluded.

### 2.3. Data Extraction and Quality Assessment

Two authors independently conducted the literature search, literature screening, and data extraction. The extracted data included the title of the study, authors, year of publication, article source, study size, total number of cases, grouping, diagnosis standard, details of methodological information, and treatment process as well as the details of the control interventions, outcomes, and adverse effects for each study. Disagreement was resolved by discussion, and consensus was reached through a third party. The methodological quality of included trials was assessed according to the Cochrane Handbook for Systematic Review of Interventions, Version 5.1.0 [13], to address the following seven criteria: random sequence generation (selection bias), allocation concealment (selection bias), blinding of participants and personnel (performance bias), blinding of outcome assessment (detection bias), incomplete outcome data (attrition bias), selective outcome reporting (reporting bias), and other sources of bias. The quality of all included trials was categorised as low, unclear, or high risk of bias (“Yes” indicates a low risk of bias, “No” indicates a high risk of bias, and “Unclear” is otherwise). Then the included trials were sorted into three categories: low risk of bias (all of the criteria were rated as having low risk of bias), unclear risk of bias (at least one item was unclear), or high risk of bias (at least one item was at a high risk of bias).

### 2.4. Data Synthesis

RevMan 5.1.0 software provided by the Cochrane Collaboration was used for data analyses. Dichotomous data were expressed as relative risk (RR) and continuous outcomes were presented as weighted mean difference (WMD), while 95% confidence intervals (CI) were calculated for both. Meta-analysis was performed if the intervention, control, and outcomes were the same or similar. The statistical heterogeneity was presented as significant when the *I* square (*I* ^2^) value exceeded 50% or *P* < 0.1. In the absence of significant heterogeneity, we pooled data using fixed effects model (*I* ^2^ < 50%); otherwise we used random effects model (*I* ^2^ > 50%) [13]. Publication bias would be explored using funnel plot analysis if a sufficient number of studies were found.

## 3. Results

### 3.1. Description of the Included Trials

**Figure 1:**
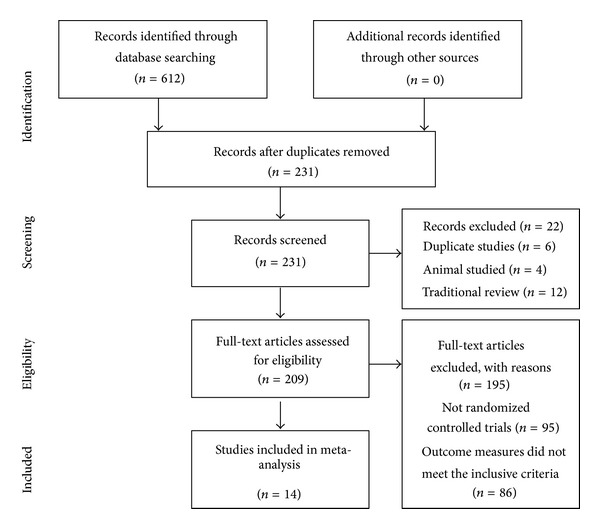
Flow chart of articles selection process.

After the primary search of the seven databases both in Chinese and English, 612 articles were retrieved: Cochrane Library (*n* = 3), PubMed (*n* = 5), Embase (*n* = 7), CNKI (*n* = 177), VIP (*n* = 142), CBMdisc (*n* = 193), and Wanfang (*n* = 85). The majority were excluded because some papers were found in more than one database and some included irrelevant titles and abstracts. Only 231 studies were retrieved. Following reviews of the titles and abstracts, several studies were excluded, and only 209 studies remained. Six trials were excluded because of duplicated publication, four trials were excluded for being animal studies, and the twelve trials were excluded for being nonclinical trials, including case reports and traditional reviews. In the end, 195 out of the remaining 209 articles were excluded based on the inclusion criteria, which left fourteen RCTs to be reviewed [14–27]. The screening process is summarised in a flow chart (Figure 1). All of the trials were conducted in China and published in Chinese. The characteristics of the fourteen RCTs are summarised in Table 1.

The fourteen RCTs involved a total number of 1180 patients with PAF. Only three trials [18, 21, 27] specified diagnostic criteria of PAF. Of those three trials, two [18, 21] used an international consensus on nomenclature and classification of AF developed by the European Society of Cardiology and the North American Society of Pacing and Electrophysiology (ESC-NASPE 2003) and Chinese Guidelines for the Management of Hypertension-2005 (CGMH-2005). The third [27] used ACC/AHA/ESC 2006 Guidelines for the Management of Patients with Atrial Fibrillation (ACC/AHA/ESC 2006). The rest of the trials [14–17, 19, 20, 22–26] only demonstrated patients with PAF diagnosis by electrocardiogram and 24-hour Holter without detailed information, and one of the trials [16] used Guidelines for the Management of Hypertension-2005 (CGMH-2005) as the diagnostic criteria for hypertension.

The interventions of all fourteen trials [14–27] included WXKL, alone or combined with Western medicine, as shown in Table 1. The controls included Western medicine alone or no medicine use. The total treatment duration ranged from two months to 24 months. Only four trials [14, 17, 20, 22] specified clinical standards of PAF. Nine of the fourteen trials [14–22] used the Pd as the main outcome measure, and seven of the fourteen trials [19, 21, 23–27] used the maintenance of sinus rhythm as the main outcome measure. Half of the included trials [16–18, 21, 23, 26, 27] described adverse effects in detail.

### 3.2. Methodological Quality of the Included Trials

The majority of the included RCTs were assessed to be of low methodological quality. According to the predefined quality assessment criteria indicated above, none of the included trials were evaluated as having a low risk of bias as shown in Table 2. Only two [22, 23] of the fourteen trials reported the methodology used to generate the allocation sequence. One [22] stated the method as odd and even numbers, and the other [23] used the table of random numbers method but without any detailed information; therefore, insufficient information was provided to allow quality assessment of the allocation method. Allocation concealment was not mentioned in every included trial. Two trials [17, 22] used the double-blind method to blind participants and personnel, but blinding of outcome assessment was not detailed in all of the trials. Only five trials [17, 18, 21, 26, 27] reported dropout or withdrawal and ten trials [14, 15, 17–19, 21, 23, 24, 26, 27] mentioned follow-up. None of the trials calculated an estimation of the pre-trial sample size, which indicated a lack of statistical power to ensure appropriate estimation of the therapeutic effect. Selective reporting was generally unclear in the trials due to the inaccessibility of the protocol. The results of the assessment of risk of bias are presented in Table 2.

### 3.3. Effects of the Interventions

#### 3.3.1. P-Wave Dispersion

**Figure 2:**
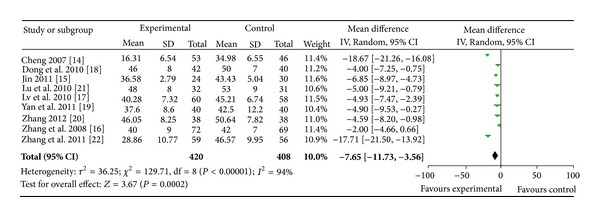
Analysis of P-wave dispersion. Forest plot of comparison: WXKL combined with Western medicine treatment group versus Western medicine treatment group.

Nine trials [14–22] used the reduction of Pd as an outcome measure. No significant difference in Pd before treatment was observed between the WXKL, alone or combined with Western medicine, group (experimental group) and Western medicine group (control group). This allowed for a comparison of Pd value of the two groups after treatment. Trial results for the nine independent trials were not homogeneous, Chi^2^ = 129.71, df = 8, (*P* < 0.00001); *I* ^2^ = 94%, requiring the use of the random effects model for statistical analysis. The Pd after WXKL, alone or combined with Western medicine, treatment was lower than Western medicine treatment. The meta-analysis demonstrated a significant difference between the two groups for each of the three criteria outcome measures (MD: −7.65 [−11.73, −3.56]; *P* = 0.0002) (Figure 2).

#### 3.3.2. Maintenance Rate of Sinus Rhythm

**Figure 3:**
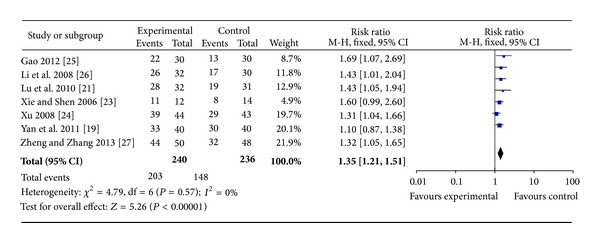
Analysis of maintaining sinus rhythm rate after six months of treatment. Forest plot of comparison: WXKL combined with Western medicine treatment group versus Western medicine treatment group.

Seven trials [19, 21, 23–27] used the maintenance rate of sinus rhythm at six months following treatment as an outcome measure. These seven trials compared the combination of WXKL plus Western medicine with Western medicine alone. Trial results for the seven independent trials were homogeneous, Chi^2^ = 4.79, df = 6, (*P* = 0.57); *I* ^2^ = 0%, requiring the use of the fixed effects model for statistical analysis. The rate of maintenance of sinus rhythm in the WXKL combined with Western medicine group (experimental group) was greater than that of the Western medicine group (control group). The rates of maintenance of sinus rhythm in the two groups were 84.6% and 62.7%, respectively. The meta-analysis showed that there was a significant beneficial effect in the combination group compared with the Western medicine alone group (RR 1.35; 95% CI [1.21, 1.51]; and *P* < 0.00001) (Figure 3).

### 3.4. Sensitivity Analysis, Subgroup Analysis, and Publication Bias

To examine the stability of the results, the fixed effects model was used to perform meta-analysis of Pd instead of the random effects model. The random effects model was used instead of the fixed effects model to analyse the maintenance of sinus rhythm. A significant difference was observed in the Pd of the two groups for the three criteria outcome measures (MD: −7.74 [−8.73, −6.75]; *P* < 0.00001). There was also a significant difference between the WXKL combined with Western medicine group and the Western medicine alone group in the maintenance of sinus rhythm (RR 1.32; 95%CI [1.18, 1.47]; and *P* < 0.00001). Given that the results of the two methods were consistent, stability was considered to be sufficient. The number of trials was too small to conduct analysis of subgroup analysis and also failed to perform funnel plot to detect publication bias.

### 3.5. Adverse Effects

Seven out of the included trials [16–18, 21, 23, 26, 27] described adverse effects in detail. One trial [16] mentioned adverse effects in both groups with one case of dry mouth and nausea, and two cases of sinus bradycardia in the WXKL group, two cases of dizziness and nausea, two cases of sinus bradycardia, one case of II atrioventricular block, and one case of Q-T interval prolongation in the amiodarone group. Three trials [17, 26, 27] mentioned specific symptoms, including vomiting, nausea, sinus bradycardia, Q-T interval prolongation, hyperthyroidism, stomach discomfort, and cough in the WXKL combined with amiodarone group and vomiting, nausea, sinus bradycardia, and hyperthyroidism in the amiodarone group. These side effects may be related to the adverse effect of amiodarone. One trial [23] reported adverse effects in the WXKL combined with irbesartan group including sinus bradycardia, mildly abnormal thyroid function, stomach discomfort, and fatigue. None of the adverse events were serious. Two trials [18, 21] reported adverse effects in the WXKL combined with fluvastatin group, including mildly abnormal liver function, which may be an adverse effect of fluvastatin. In total, the incidence of adverse reactions was lower in the treatment group compared with the control group.

## 4. Discussion

This systematic review included fourteen RCTs with a total of 1180 participants. The main findings of this systematic review were that WXKL combined with Western medicine demonstrated the potential effect of lowering Pd and improving the maintenance of sinus rhythm when compared with Western medicine alone or no treatment. WXKL is an effective treatment for patients with PAF. However, due to the poor methodological quality of the included studies, the evidence remains weak. Thus, the available data are not adequate to draw a definite conclusion of the efficacy of WXKL for PAF, but they provide some encouraging evidence of the effect of WXKL for the treatment of PAF. Nine trials used the reduction of Pd as an outcome measure and trial results for the nine independent trials were not homogeneous. The main source of the heterogeneity may have several aspects. The treatment courses are not the same, the longest is 24 month, while the shortest is 2 month. The dosage and the time of WXKL the patients used were also consistent. Because of different diagnosis standards, different doctors' diagnosis can make a big difference. So the clinical and methodological source made the heterogeneity of statistics.

AF is one of the most common chronic arrhythmia conditions associated with an adverse prognosis. Many studies show that subjects with AF have markedly reduced survival compared with subjects without AF and that AF is independently associated with a 50% to 90% increase in the risk of death. Therefore, the effective treatment and prevention of AF has important clinical significance [28]. Many studies have shown that atrial electrical remodelling and structural remodelling of AF are the main mechanism for precipitating and maintaining sinus rhythm. Electrical remodelling could shorten atrial refractoriness and contributes to an increase in the stability of AF. Atrial structural remodelling occurs as a result of heart failure and other underlying cardiovascular diseases [29, 30]. The ionic mechanisms underlying this arrhythmogenic process have been elucidated by a number of patch clamp experiments in isolated atrial cells from animal models and patients in chronic AF. The most important impact of AF on the ion channels was a marked reduction in the L-type Ca^2+^ current [31].

AF is a commonly encountered arrhythmia that occurs in patients after coronary artery bypass surgery (CABG). A study has shown that the preoperative signal averaged ECG (SAECG) Pd was the best predictor of AF after CABG [32]. Pd is an appealing marker for predicting the risk of developing atrial fibrillation. The increase of Pd is a good predictor of the occurrence of PAF and it is also an important electrophysiological cause of AF [33]. However, a study showed that this technique has limited sensitivity and specificity because there is overlap between the wide range of values of Pd, Pmax, and Pmin in healthy individuals that overlaps with those of patients with increased risk for AF. This may stem from methodological issues; therefore, there is a definite need for methodological standardisation of Pd measurements [34]. Most pharmacologic therapies and electrical cardioversion for AF unsuccessfully treat the atrial electrical remodelling and structural remodelling of AF [35], so it is particularly necessary to explore traditional Chinese medicine for the treatment of PAF.

WXKL is the first antiarrhythmic Chinese medicine to be approved by the state. It is developed from the application of traditional medicine theory combined with the essence of Chinese and Western medicine theory. It composed of five main components: nardostachys chinensis batal extract, codonopsis, notoginseng, amber, and rhizoma polygonati. A study has shown that WXKL is a novel atrial elective sodium channel blocker and is effective in suppressing AF and preventing its induction [36]. From the included articles, we can see WXKL has significant effect on improving the main symptoms such as headache, dizziness, palpitations, and insomnia. This meta-analysis showed that WXKL combined with Western medicine demonstrated the potential effect of lowering Pd and improving the maintenance of sinus rhythm.

However, this systematic review has the following limitations. Firstly, the quality of the methodology of the RCTs included in this systematic review is generally low. All of the included trials claimed randomisation, but only two of them [22, 23] reported the methodology used to generate the allocation sequence. The other trials mentioned only that “patients were randomized into two groups,” indicating potential selection bias. Two trials [17, 22] mentioned the double-blind method of blinding participants and personnel but did not provide sufficient information for quality assessment, which might lead to potential performance bias and detection bias. Five of the trials [14, 15, 20, 24, 25] were conducted by a single author, which could lead to performance bias. Only five trials [17, 18, 21, 26, 27] reported dropout or withdrawal, but without the intention-to-treat analysis. And ten RCTs [14, 15, 17–19, 21, 23, 24, 26, 27] mentioned follow-up. None of trials gave a pretrial estimation of sample size, which could indicate a lack of statistical power to ensure appropriate estimation of the therapeutic effect. It is well known that the trials that are poorly designed methodologically show larger differences compared with rigorously conducted trials. Additionally, all of the trials were conducted in China and published in Chinese, leading to publication bias. All of the RCTs claimed that the positive effect of WXKL combined with Western medicine is better than Western medicine alone or no medicine. While no negative findings have been reported, we cannot eliminate the possibility of the unpublished material.

Secondly, the safety of Chinese herbal medicines is of general concern. Adverse effects were reported in seven out of the fourteen included trials [16–18, 21, 23, 26, 27]. Some adverse effects were not severe and patients spontaneously recovered without special treatment. However, some adverse effects are irreversible. In total, the incidence of adverse reactions was lower in experimental groups compared with the control groups. The other seven trials did not report any adverse effects. Due to the limited evidence provided by the eligible trials, conclusions about the safety of WXKL combined with Western medicine cannot be drawn from this study. In the future, larger-scale clinical trials with long-term follow-up are warranted to properly assess the safety of WXKL therapy.

Thirdly, publication and other biases may play an important role. We only identified and included trials published in Chinese and most of the trials are small sample with positive findings. We tried to avoid language bias and location bias, but we cannot exclude potential publication bias. During the articles assessed for eligibility, we found that the majority was excluded because of no detailed information and the quality of the methodology of the RCTs included in this systematic review is generally low. We recommend that future researchers should follow the basic guidelines for reporting clinical trials conducted with clear TCM diagnostic criteria.

In addition, the currently available antiarrhythmic drugs for PAF suffer from limited safety and efficacy, probably because they were not designed based on specific pathological mechanisms. We should clarify the main pathological mechanisms of AF, understand traditional and novel aspects of antiarrhythmic drugs in relation to these pathological mechanisms, and present potential therapeutic approaches, including restoring abnormal Ca^2+^ handling in AF, structure-based modulation of atrial-specific cardiac ion channels, and targeting atrial remodelling. Moreover, we should continue to expand the cumulative meta-analysis of future trials, especially RCTs, and choose possible predictors of PAF to provide more meaningful clinical indicators for clinicians.

In summary, there is an encouraging evidence of the effect of WXKL, alone or combined with Western medicine, on Pd and the maintenance of sinus rhythm in patients with PAF. After treatment with WXKL, there is a significant reduction in Pd in patients with PAF, and the maintenance of sinus rhythm is significantly improved. Due to the poor quality of experimental design and methodology, the evidence remains weak. More rigorous RCTs with strong design and high methodological quality will be needed to present a high level of evidence for the effectiveness of WXKL in treating PAF.

## Figures and Tables

**Table 1:** Characteristics and methodological quality of the included studies.

Study	Sample size (treatment/control)	Diagnosis standard	Complications	Intervention	Control	Treatment course (month)	Clinical standards	Outcome measure
Cheng 2007 [14]	99 (53/46)	Diagnostic criteria for PAF (unclear)	PAF	WXKL 9 g tid + control	Enteric-coated aspirin	3	Clinical guideline of new drugs for TCM (1995)	Pd

Jin 2011 [15]	60 (30/30)	Diagnostic criteria for PAF (unclear)	PAF	WXKL 9 g tid	Conventional therapy (no detailed information)	6	Unclear	Pd

Zhang et al. 2008 [16]	141 (72/69)	Diagnostic criteria for PAF (unclear) CGMH (2005)	Elderly hypertension and PAF	WXKL 9 g qd	Amiodarone	2	24 h Holter	Pd, ADR

Lv et al. 2010 [17]	120 (60/60)	Diagnostic criteria for PAF (unclear)	Elderly PAF	WXKL 9 g tid + control	Amiodarone	12	Clinical guideline of new drugs for TCM (1995)	Pd, ADR

Dong et al. 2010 [18]	86 (42/40)	ESC-NASPE (2003) CGMH (2005)	Hypertension and PAF	WXKL 9 g tid + fluvastatin	Conventional therapy (no detailed information)	12	Unclear	Pd, ADR

Yan et al. 2011 [19]	80 (40/40)	Diagnostic criteria for PAF (unclear)	PAF	WXKL 5 g qd + control	Amiodarone	12	Unclear	Pd, maintenance rate of sinus rhythm

Zhang 2012 [20]	76 (38/38)	Diagnostic criteria for PAF (unclear)	PAF	WXKL 5 g tid + control	Amiodarone	6	National integrative arrhythmia prevention research symposium revised standard	Pd

Lu et al. 2010 [21]	68 (34/34)	ESC-NASPE (2003) CGMH (2005)	Elderly hypertension and PAF	WXKL 9 g tid + fluvastatin	Conventional therapy (no detailed information)	6	Unclear	Pd, maintenance rate of sinus rhythm, ADR

Zhang et al. 2011 [22]	115 (59/56)	Diagnostic criteria for PAF (unclear)	Elderly DHF and PAF	WXKL 9 g tid	Conventional therapy (no detailed information)	3	Antiarrhythmic drug therapy recommendations (2001)	Pd

Xie and Shen 2006 [23]	26 (12/14)	Diagnostic criteria for PAF (unclear)	PAF	WXKL 9 g tid + irbesartan	Amiodarone	6	Unclear	Maintenance rate of sinus rhythm, ADR

Xu 2008 [24]	87 (44/43)	Diagnostic criteria for PAF (unclear)	PAF	WXKL 9 g tid + valsartan	Amiodarone	24	Unclear	Maintenance rate of sinus rhythm

Gao 2012 [25]	60 (30/30)	Diagnostic criteria for PAF (unclear)	Hypertension and PAF	WXKL 9 g tid + control	Valsartan	12	Unclear	Maintenance rate of sinus rhythm

Li et al. 2008 [26]	62 (32/30)	Diagnostic criteria for PAF (unclear)	PAF	WXKL 9 g tid + control	Amiodarone	6	Unclear	Maintenance rate of sinus rhythm, ADR

Zheng and Zhang 2013 [27]	100 (50/50)	ACC/AHA/ESC (2006)	PAF	WXKL 9 g tid + control	Amiodarone	9	Unclear	Maintenance rate of sinus rhythm, ADR

**Table 2:** Quality assessment of the included randomized controlled trials.

Included trials	Sequence generation	Allocation concealment	Blinding of participants personnel	Blinding of outcome assessors	Incomplete outcome data	Selective outcome reporting	Other sources of bias	Risk of bias
Cheng 2007 [14]	Unclear	Unclear	Unclear	Unclear	Unclear	No	Unclear	High

Jin 2011 [15]	Unclear	Unclear	Unclear	Unclear	Unclear	No	Unclear	High

Zhang et al. 2008 [16]	Unclear	Unclear	Unclear	Unclear	Unclear	No	Unclear	High

Lv et al. 2010 [17]	Unclear	Unclear	Double-blind method	Unclear	Yes	No	Unclear	Unclear

Dong et al. 2010 [18]	Unclear	Unclear	Unclear	Unclear	Yes	No	Unclear	High

Yan et al. 2011 [19]	Unclear	Unclear	Unclear	Unclear	No	No	Unclear	High

Zhang 2012 [20]	Unclear	Unclear	Unclear	Unclear	Unclear	No	Unclear	High

Lu et al. 2010 [21]	Unclear	Unclear	Unclear	Unclear	Yes	No	Unclear	High

Zhang et al. 2011 [22]	Odd and even numbers	Unclear	Double-blind method	Unclear	Unclear	No	Unclear	Unclear

Xie and Shen 2006 [23]	Table of random number	Unclear	Unclear	Unclear	Unclear	No	Unclear	Unclear

Xu 2008 [24]	Unclear	Unclear	Unclear	Unclear	Unclear	No	Unclear	High

Gao 2012 [25]	Unclear	Unclear	Unclear	Unclear	Unclear	No	Unclear	High

Li et al. 2008 [26]	Unclear	Unclear	Unclear	Unclear	Yes	No	Unclear	High

Zheng and Zhang 2013 [27]	Unclear	Unclear	Unclear	Unclear	Yes	No	Unclear	High
